# The development of activatable lytic peptides for targeting triple negative breast cancer

**DOI:** 10.1038/cddiscovery.2017.37

**Published:** 2017-07-17

**Authors:** Hui Zhao, Xuan Qin, Dan Yang, Yanhong Jiang, Weihao Zheng, Dongyuan Wang, Yuan Tian, Qisong Liu, Naihan Xu, Zigang Li

**Affiliations:** 1 Department of Chemical Biology, School of Chemical Biology and Biotechnology, Shenzhen Graduate School of Peking University, Shenzhen, China; 2 Department of Pharmacy, School of Life Science and Engineering, Southwest Jiaotong University, Chengdu, China; 3 Shenzhen Key Lab of Tissue Engineering, The Second People's Hospital of Shenzhen, Shenzhen, China; 4 Key Lab in Healthy Science and Technology, Division of Life Science, Tsinghua University Shenzhen Graduate School, Shenzhen, China

## Abstract

Cytolytic peptides are an emerging class of promising cancer therapeutics shown to overcome drug resistance. They eliminate cancer cells via disruption of the phospholipid bilayer of cell membranes, a mechanism that differentiates it from traditional treatments. However, applications of lytic peptides via systematic administration are hampered by nonspecific toxicity. Here, we describe activatable, masked lytic peptides that are conjugated with anionic peptides via a cleavable linker sensitive to matrix metalloproteinases (Ac-w-*β*A-e_8_-XPLG*LAG-klUklUkklUklUk-NH_2_; lower case letters in the sequences represent D-amino-acids, U=Aib, *α*-aminoisobutyric acid, *cleavage site). The peptides were activated upon being introduced into the triple negative breast cancer cell line MDA-MB-231, which overexpresses secreted matrix metalloproteinases, to selectively cleave the peptide linker. Our results indicate that the activatable design could be applied to improve the targeting ability of lytic peptides.

## Introduction

Cytolytic peptides typically have both cationic and amphiphilic properties, resulting in the lysis of cells in a nonspecific manner. In general, these cytolytic peptides induce cell lysis by associating with the plasma membrane via electrostatic interactions. These interactions disrupt the membrane and form pores, leading to rapid necrotic cell death.^1^ Conventional cancer chemotherapies develop drug resistance quickly, and novel therapeutics are in urgent need. Various new classes of promising cancer therapeutics have emerged recently, including cytolytic peptides^2^ and oncolytic viruses.^3^ For example, melittin, a major constituent of bee venom, raised interest as a potential anticancer reagent in recent years.^4,5,6^

The activity of cytolytic peptides is largely dominated by their biophysical properties, including charges and secondary structures. For example, Wimley *et al.*^7,8,9,10^ reported that tuning the conformation of cytolytic peptides could modulate their activity. We also recently developed an efficient cytolytic peptide derived from BH3-only domains using rational design including sequence alignment, conformation constraint, and charge tuning.^11^

The main limitation of cytolytic peptides is their nonspecific cytotoxicity. To address this issue, Kawakami *et al.* conjugated lytic peptides with targeting sequences that bind specific receptors known to be overexpressed in tumour cells.^12,13,14,15^ In addition, different formulations were utilized to test delivery of lytic peptides, such as the use of hybrid peptide-hydrogel complexes as effective delivery formulations.^16^ Different nanoparticles were also applied for delivery of melittin, including polyethylene perfluorocarbon,^17^ poly(lactic-co-glycolic acid) nanoparticles,^18^ glycol-stabilized lipid disks,^19^ quantum dots,^20^ and core-shell lipid nanoparticles,^21^ resulting in a reduction of melittin haemolytic activity and an increase in targeted accumulation in the tumour.

To achieve targeted delivery of cytolytic peptides to tumours, unique properties of the tumour microenvironment must be exploited. In addition to properties such as the excessive leakiness of tumour vasculature, poor lymphatic drainage, hypoxic and acidic conditions, upregulated enzymes in tumour cells were also widely used for targeted delivery. For example, fibroblast-activation protein-*α* (FAP-*α*)^22^ and urokinase-type plasminogen activator (uPA)^23^ have been used to achieve targeted delivery of melittin. Matrix metalloproteinases (MMPs) are another example of a class of secreted enzymes known to be overexpressed in specific cancer cells, including the triple negative breast cancer cell line, MDA-MB-231.^24^ Patients with triple negative breast cancer always have a poor prognosis. These enzymes play a vital role in matrix degradation, inflammation, and tumour cell migration.^25^ Thus, MMPs are of great interest in terms of tumour-specific drug delivery.^26^ One example of this is the development of a melittin/avidin conjugate^27^ and a melittin-MMP-2-LAP^28^ recombinant adenovirus to increase MMP-2-specific targeting. Additionally, a MMP-9-activated melittin prodrug was recently tested in combination with perfluorocarbon nanoparticles,^29^ which showed a significant enhancement in *in vivo* efficacy.

Tsien *et al.* reported the development of an activatable cell-penetrating peptide (ACPP)^30^ that consists of a cationic CPP coupled with complimentary anionic peptides via a cleavable linker. This cleavable linker enhances the pharmacokinetic properties of the peptides and avoids nonspecific cytotoxic effects associated with CPPs.^31^ The ACCPs developed from this study have been used either independently or complexed with nanoparticles in imaging studies^32,33,34^ and therapeutic interventions.^35,36^

Here, we report the development of activatable, masked lytic peptides that take advantage of over-expressed MMPs to elicit selective cytotoxicity against the triple negative breast cancer cell line, MDA-MB-231. The derived lytic peptides show limited nonspecific toxicity and improved targeting ability. Our results indicate that this activatable strategy could be applied to achieve systematic administration of lytic peptides.

## Results

### Design, synthesis, and initial evaluation of lytic peptides

**Figure 1:**
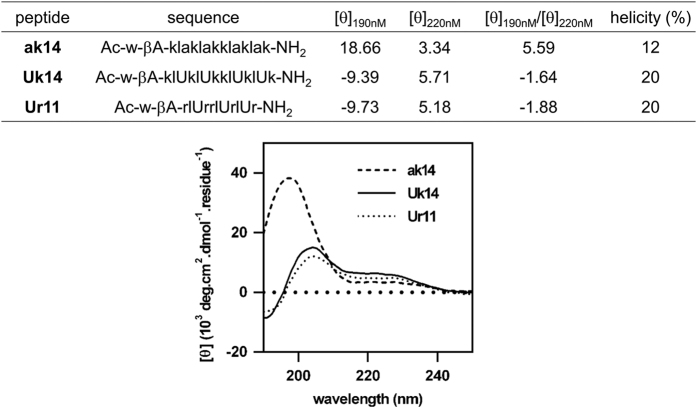
Helical conformation of peptides. Circular dichroism spectra, molar elipticities (10^3^ deg cm^2^/dmol per residue) at 220 nm, ratios of elipticites at 190/220 nm, and calculated helicity (%) for ak14, Uk14 and Ur11 peptides in 10 mM phosphate buffer, pH 7.4, 298 K. The *α*-helices display two separate negative maximum signals at both 222 and 208 nm as well as another positive signal at 195 nm. Small letters in the sequences indicate D-amino-acids. U=Aib, *α*-aminoisobutyric acid.

To create effective short lytic peptides, we began with the pro-apoptotic peptide (klaklak)_2_.^37^ A D-Trp was attached to each peptide for concentration determination using a *β*-Ala as the linker. This is a standard cationic, amphiphilic *α*-helical peptide that functions by disrupting the mitochondrial membrane, resulting in mitochondria-dependent cell-free apoptosis. Conjugation of this peptide with a targeting agent could be used to induce targeted apoptosis in tumour cells.^37,38,39^ However, ak14 (the number refers to the length of the active sequence) showed only moderate efficiency at targeting MDA-MB-231 cells, as measured in 3-(4,5-dimethyl-2-thiazolyl)-2,5-diphenyl-2H-tetrazolium bromide assays. Previous reports suggest that the efficiency of lytic peptides is closely tied to their secondary structure. Thus, we proceeded to substitute alanine residues with *α*-aminoisobutyric acid (Aib, U), an amino acid known to stabilize helical structures.^40,41,42^ Indeed, the improved peptide, Uk14, showed a higher helical content (20%) and significantly enhanced cytotoxicity toward MDA-MB-231 cells (IC_50_=4.5 *μ*M) compared to ak14 (helicity=12%, IC_50_>30 *μ*M, Figure 1, Supplementary Table S1 and Supplementary Figure S1), which could be explained by the introduction of *α*-methyl groups. We also showed that truncations (Uk11) or elimination of secondary structural amphiphilicity (lUk) of Uk14 result in decreased efficiency. (Supplementary Table S1 and Supplementary Figure S1).

We then replaced D-Lys residues in the Uk14 peptide with the membrane-activating amino acid, D-Arg. This resulted in the generation of peptide Ur14. However, no significant improvements were observed with this Ur14 peptide (Supplementary Table S1 and Supplementary Figure S1). Furthermore, the truncation of peptide Ur14 to generate peptide Ur11 showed no change in activity. A further truncated peptide, Ur10, exhibited greatly diminished activity towards MDB-MA-231 cells (Supplementary Table S1 and Supplementary Figure S1). Both peptides, Uk14 and Ur11, showed helical conformation in water (Figure 1) and displayed broad spectrum growth inhibition towards the breast cancer cell line MCF-7, and the cervical cancer cell line HeLa and SK-OV-3, a human ovary adenocarcinoma cell line. (Table 1 and Supplementary Figure S2).

### Mechanism of action of lytic peptides

**Figure 2:**
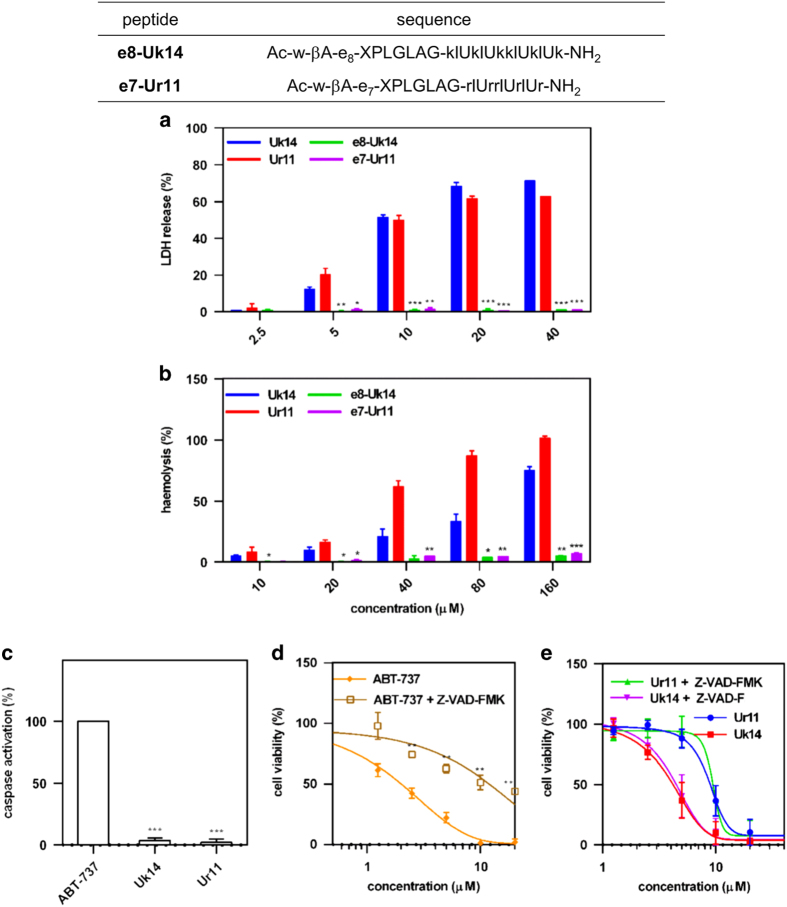
Reduced toxicity of masked lytic peptides. (**a**) Lactate dehydrogenase release of MDA-MB-231 cells following treatment with Uk14, Ur11, e8-Uk14, e7-Ur11 (4 h treatment). (**b**) Haemolysis assays for Uk14, Ur11, e8-Uk14, e7-Ur11 in red blood cells. (1 h treatment). (**c**) Caspase activation in MDA-MB-231 cells following treatment with Uk14 (5 *μ*M, 4 h treatment), Ur11 (10 *μ*M, 4 h treatment) and ABT-737 (10 *μ*M, 4 h treatment). Action of ABT-737 (**d**) or peptides Ur11 and Uk14 (**e**) with or without Z-VAD-FMK on MDA-MB-231 (24 h treatment). Small letters in the sequences indicate D-amino-acids. U=Aib, *α*-aminoisobutyric acid. Error bars represent the standard deviation from more than at least two independent experiments. *P<0.05, **P<0.01, ***P<0.001 compared with corresponding unmasked peptides-treated cells (Uk14 *versus* e8-Uk14, Ur11 *versus* e7-Ur11) in **a** and **b**, ABT-737-treated cells in **d** and cells without treatment of Z-VAD-FMK in **d** and **e**.

After proving that these peptides were active, we aimed to understand their mechanism of action. Lactate dehydrogenase release assays revealed that treatment of cells with peptides Uk14 and Ur11 led to a loss of membrane integrity (Figure 2a). Consistent with the high levels of lactate dehydrogenase release observed upon treatment with peptides Uk14 and Ur11, these peptides showed strong haemolytic activity (Figure 2b). Thus, the direct applications of these peptides are limited. Furthermore, caspase-3/7 activation, which is an indicator of apoptosis, was not observed in Uk14 or Ur11 peptide-treated MDA-MB-231 cells, in contrast to what was observed with treatment of ABT-737 (Figure 2c). The addition of caspase inhibitor, Z-VAD-FMK, did not block the cytotoxic effects of Uk14 or Ur11 in MDA-MB-231 cells but did have an inhibitory effect on ABT-737 (Figures 2d and e). These results suggested that peptides Uk14 and Ur11 induced cell death primarily through cell membrane disruption mechanisms,^1,11^ rather than induction of apoptosis via caspase activation.

### Activatable modifications of lytic peptides

Next, we conjugated a poly-glutamate mask to Uk14 (Ac-w-*β*A-e_8_-XPLG*LAG-klUklUkklUklUk-NH_2_) and Ur11 (Ac-w-*β*A-e_8_-XPLG*LAG-rlUrrlUrlUr-NH_2_) peptides using the MMP-specific cleavage sequence, XPLG*LAG, (X=6-aminohexanoyl, *cleavage site) to create peptides e8-Uk14 and e7-Ur11 (Figure 2; *n* in en represents the number of glutamic acids; (−) indicates the linker; poly-glutamate was linked to the N-terminus of parent lytic peptides via the MMP-specific cleavage sequence). Unlike the unmodified peptides, these modified ones exhibited diminished membrane disruption and haemolysis (Figures 2a and b) and no obvious cytotoxicity against the non-tumourigenic cell line, HEK293T (Supplementary Figure S3).

**Figure 3:**
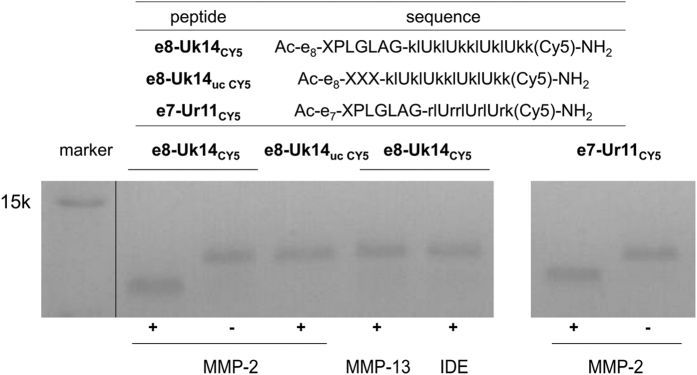
Selectively unmasked lytic peptides upon protease cleavage of a linker selective for matrix metalloproteinases. Enzyme cleavage of Cy5-labelled e8-Uk14 and e7-Ur11. Peptides (3 mM) were incubated with activated enzymes (50 nM) in cleavage buffer for 30 min at 310 K. Cy5 was labelled on the D-Lys in peptides. Small letters in the sequences indicate D-amino-acids. X=6-aminohexanoyl. U=Aib, *α*-aminoisobutyric acid. IDE, insulin degrading enzyme. MMP, matrix metalloproteinase. uc, uncleavable.

Furthermore, masked peptides were able to be selectively cleaved by MMP-2 (Figure 3) to release the active parts, which were membrane disruptive. While the peptide e8-Uk14_uc CY5_, (uc=uncleavable) whose cleavable linker PLG*LAG was replaced by uncleavable 2X (X=6-aminohexanoyl, 2X=two continuous X), showed no response to MMP treatment. Collectively, these results indicate that (1) a poly-glutamate mask decreases a peptides’ ability to disrupt the cell membrane and reduces its nonspecific toxicity; and (2) MMP can cleave the peptide linker and release the lytic peptides Uk14 and Ur11.

### *In vivo* distribution of activatable lytic peptides

**Figure 4:**
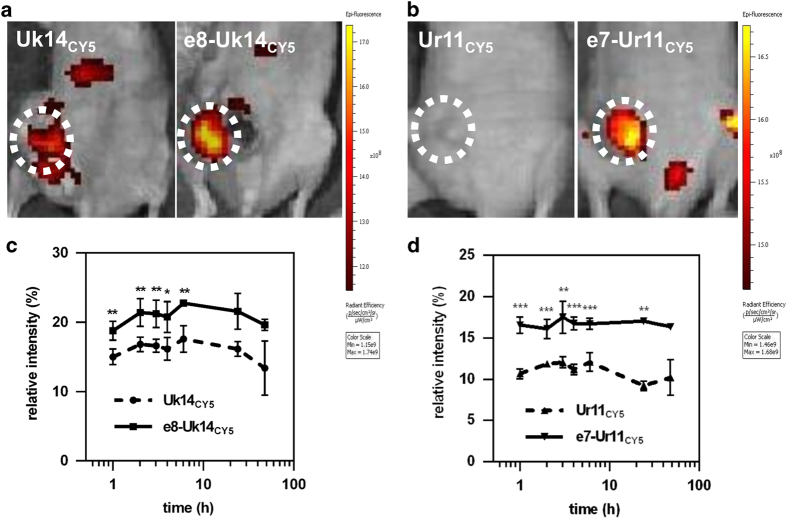
Real-time fluorescence imaging showed the *in vivo* distribution of lytic peptides in the MDA-MB-231 bearing mice. (**a** and **b**) *In vivo* imaging of mice treated with Cy5-labelled Uk14, e8-Uk14, (**a**) Ur11 and e7-Ur11 peptides (**b**) (100 *μ*l, 50 *μ*M) images were taken 6 h following systemic administration. Uk14_CY5_ and e8-Uk14_CY5_ shared a scale bar; Ur11_CY5_ and e7-Ur11_CY5_ shared one. Tumours are indicated by dashed circles. Luminance in circles denotes the intensity of fluorescence. (**c** and **d**) Time-tumour fluorescence/chest and abdomen fluorescence ratio curve of Cy5-labelled Uk14, e8-Uk14, (**c**) Ur11 and e7-Ur11 peptides (**d**) fluorescence in manually set regions of interest was quantified by Living Image. Error bars represent the standard deviation from at least two independent experiments. (*n*=2) *P<0.05, **P<0.01, ***P<0.001 compared with corresponding unmasked peptides-treated mice.

To evaluate the *in vivo* distribution of these peptides, 100 *μ*l (50 *μ*M) of Cy5-labelled peptides Uk14, Ur11, e8-Uk14 and e7-Ur11 were injected *via* the tail vein of mice bearing an MDA-MB-231 tumour (approximately 50 mm^3^).^31^ Modified peptides e8-Uk14 and e7-Ur11 showed significantly higher accumulation in tumours compared to unmodified peptides (Figures 4a and b). The moderate accumulation of unmodified lytic peptides in tumours could be explained by the tumour targeting potential of the R/KXXR/K epitope.^43^ According to the time-tumour fluorescence/chest and abdomen fluorescence ratio curve (Figures 4c and d), peptides e8-Uk14 and e7-Ur11 showed consistently higher tumour accumulation levels compared to unmodified peptides. This indicates that the activatable modification of peptides could increase their targeting ability. Tumour accumulation of these peptides reaches maximum levels after approximately 2 h and is observed to persist for a minimum of 24 h. This long persistence time could be explained by the proteolytic resistant nature of Uk14 and Ur11. In summary, activatable lytic peptides showed an efficient tumour targeting ability. Additionally, the ability to label these peptides could enable them to act as a tool for tumour imaging.

**Figure 5:**
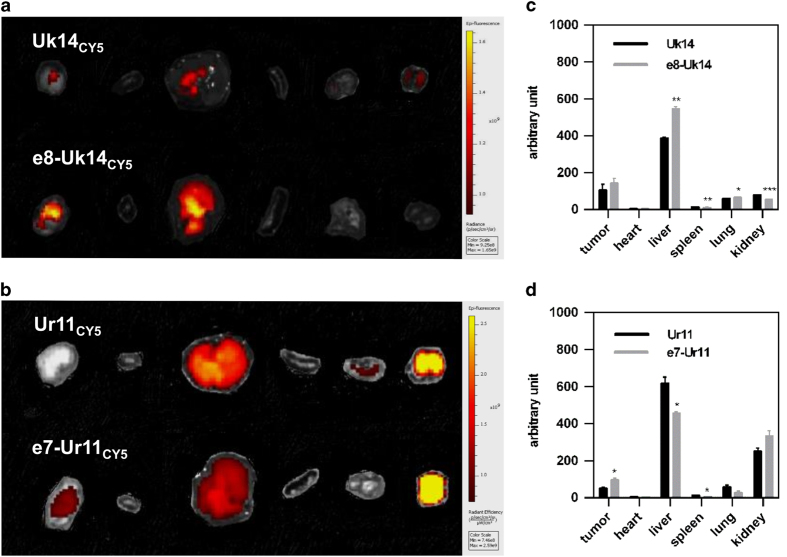
Tissue distribution of lytic peptides in MDA-MB-231 bearing mice. (**a** and **b**) Imaging of exposed main organs that were excised at 6 h after administration of Cy5 labelled peptides. (100 *μ*l, 50 *μ*M) (from left to right: tumour, heart, liver, spleen, lung, and kidney). (**c** and **d**) Corresponding fluorescence of exposed main organs that were excised at 6 h after administration. Fluorescence in manually set regions of interest was quantified by Living Image. Error bars represent the standard deviation from two independent experiments. (*n*=2) *P<0.05, **P<0.01, ***P<0.001 compared with corresponding unmasked peptides-treated mice.

At 6 h post-peptide injection, we quantified peptide accumulation in different critical organs (tumour, heart, liver, spleen, lung, and kidney; Figure 5). Uptake levels of e8-Uk14 and e7-Ur11 in tumours were higher compared to UK14 and Ur11. Notably, peptides were observed to accumulate primarily in the liver, lung and kidney, observations commonly made in peptide therapeutics.^31^ This was especially true for Ur11, which accumulated at much higher levels in the kidney compared to Uk14. In addition, compared to Uk14, e8-Uk14 showed higher accumulation in the liver, but lower accumulation in the kidney. This observation could be explained by differences in clearance rates.^31^ We conclude that activation design result in increased tumour accumulation compared to unmodified lytic peptides.

### *In vivo* activities of activatable lytic peptides

We next tested acute toxicity of the cleavable lytic peptide. Using a dose of 40 mg/kg (200 *μ*l, 4 mg/ml), we observed that e7-Ur11 caused death (one in three survived), while e8-Uk14 showed no acute toxicity (Supplementary Table S2). This could be due to nonspecific proteolysis and subsequent release of the lytic components of e7-Ur11. We note that higher accumulation levels of these peptides in critical organs, such as the kidney, could also cause harmful effects to nude mice. (Supplementary Figure S4) In view of the higher tumour site accumulation and lower acute toxicity of e8-Uk14 than e7-Ur11, we chose e8-Uk14 for further evaluation.

**Figure 6:**
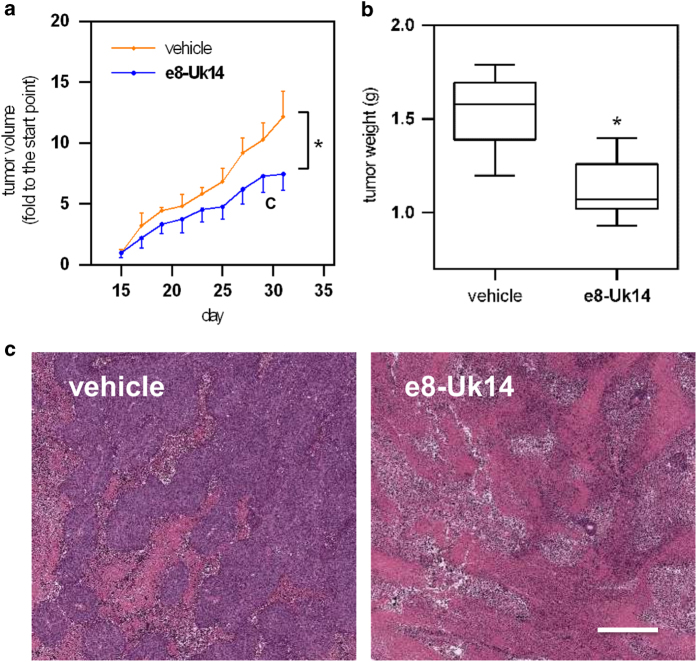
*In vivo* evaluation of the effect of e8-Uk14 on the inhibition of MDA-MB-231 tumour growth. Mice were administered via intravenous injection vehicle control solvent (saline) and peptide at the dose of 20 mg/kg in every other day over a period of 2 weeks. (**a**) Tumour size of MDA-MB-231 xenografts. Tumour size was measured by calliper measurements over a period of 2 weeks (*n*=5). Error bars represent the standard deviation. (**b**) Tumour weights of MDA-MB-231 xenografts. Mice were killed, and tumours were resected after the final injection. Error bars represent maxima and minima; boxes represent the upper and lower quartiles and the median±standard deviation. (*n*=5). (**c**) Haematoxylin-eosin staining of tumour cross-sections from mice treated with e8-Uk14 and control. Scale bar, 100 *μ*m. *P<0.05 compared with vehicle peptides-treated mice.

To study the anticancer activity of activatable peptides *in vivo*, we injected nude mice with human breast cancer cells (MDA-MB-231), followed by administration of the e8-Uk14 peptide or vehicle control. The typical increase in tumour volume and weight after introduction of these cancer cells to mice was slowed following a 14-day treatment with the activatable e8-Uk14 peptide (*n*=5) compared with vehicle control treatment (*n*=5, 20%, *P<0.05; Figures 6a and b). Histological staining (haematoxylin-eosin staining) showed that while cells were densely packed in the tumour tissue of control mice, the cell density was significantly reduced in the tumour tissue of e8-Uk14-treated mice. (Figure 6c).

## Discussion

Lytic peptides have shown a remarkable ability to eliminate cancer cells via a different mechanism than traditional treatments. Conformational tuning has been proven to generate gain of function variations to modulate the activities of lytic peptides. Starting from an amphiphilic, mitochondrial membrane disruptive sequence, efficient lytic peptides were evolved by conformation constraint, mutations, and truncations, which exhibited cytotoxicity against a broad spectrum of cancer cell lines, including the triple negative breast cancer cell line MDA-MB-231, the oestrogen receptor-positive breast cancer cell line MCF-7, and the cervical cancer cell lines HeLa and SK-OV-3, a human ovary adenocarcinoma cell line. Topological amphiphilicity of sufficient length is strictly required for the cellular activities of these lytic peptides. The lytic peptides induce cell death in an apoptosis-independent manner by causing disruption of the cell membrane, and they showed haemolytic activity. In the case of peptide Uk14, conformational constraints lead to mechanistic changes, where an apoptotic sequence turned into necrotic peptides via mere constraining of the conformation. Such mechanistic changes have also been observed in the case of (klaklak)_2_ based amphiphiles,^44^ which are achieved via lipid conjugation. These results suggest that the action of a peptide is not solely determined by the peptide sequence but is a result of the entirety of its physicochemical properties.^45^ These results confirmed that conformational constraint could be applied to optimize the anticancer activity of lytic peptides.

One of the main limitations of the applications of lytic peptides is their nonspecific cytotoxicity. To provide an avenue to address this issue, a masking sequence was attached to lytic peptides via an MMP responsive sequence based on the design of activatable cell penetrating peptides. According to the *in vitro* results, the poly-glutamate mask decreases a peptides’ ability to disrupt the cell membrane and reduces its nonspecific toxicity. The masked peptides are selectively cleaved by MMP-2 to release the lytic peptides. These results provide a solid basis for further *in vivo* evaluation of these masked lytic peptides.

MMP is known to be overexpressed in specific cancer cells, including the triple negative breast cancer cell line MDA-MB-231, for which there is currently no targeted therapy. In MDA-MB-231 xenografts, systematically administered, activatable, masked lytic peptides showed increased tumour accumulation compared to unmodified lytic peptides. Tumour accumulation of these lytic peptides was still observed after 48 h, which suggested that these peptides could be used for tumour imaging. In addition, one of the masked lytic peptides showed anti-tumour activities *in vivo* via systematic administration. Notably, the activatable design could be transferred to other diseases, where a specific enzyme is identified in pathological sites. However, acute toxicity was still observed for the masked lytic peptides, which might be attributable to nonspecific cleavage. Additionally, higher accumulation levels of these peptides in critical organs, such as the liver and kidney, was noted, which could also cause harmful effects to nude mice. Accordingly, further targeted modifications should be conducted to improve *in vivo* pharmacodynamic properties and anti-tumour efficacies.

Through peptide conformation tuning, mutations, and truncations, we have developed two short lytic peptides that show wide-spectrum inhibition of cancer cell growth. Modifications to make these peptides activatable reduced their *in vitro* nonspecific toxicity and increased their specific targeting ability. These activatable lytic peptides showed moderate tumour inhibition activity *in vivo* upon systematic administration. This activatable peptide strategy is further proven by this study as a potential masking method for other bio-relevant peptides. The peptides developed in this work could be further optimized to enable their use in tumour imaging and therapeutic interventions.

## Materials and methods

### Materials

All solvents and reagents used for solid phase peptide synthesis were purchased from commercial suppliers including GL Biochem (Shanghai, China), Hanhong Chemical (Shanghai, China), Energy Chemical (Shanghai, China), and Tenglong Logistics (Shenzhen, China) were used without further purification unless otherwise stated.

### Peptide synthesis

Peptides were synthesized on Rink-amide-MBHA resin using manual Fmoc/tButyl solid-phase peptide synthesis. Coupling reactions were performed using 2-(1H-6-chlorobenzotriazol-1-yl)-1,1,3,3-tetramethyluronium hexafluorophosphate for 3 h with N_2_ bubbling. Cy5 labelling was performed in solution with the solution of Cy5-N-hydroxysuccinimide ester in NaHCO_3_ (10 mM, pH=8.0) overnight. Final resins were treated with 95% (v/v) trifluoroacetic acid/triisopropylsilane/H_2_O (95 : 2.5 : 2.5) for 2 h. After air removal of most of the TFA, products were triturated with hexane/diethyl ether (1 : 2), dissolved in CH_3_CN/H_2_O (1 : 1). Crude peptides were purified on reverse phase high-performance liquid chromatography (Agilent (Santa Clara, CA, USA) Zorbax SB-Aq: 4.6×250 mm, 220 and 254 nm) and confirmed by Shimadzu (Kyoto, Japan) liquid chromatography-mass spectrometry 2020 mass spectrometer equipped with Agilent Zorbax SB-Aq column. Characterization of peptides is shown in Supplementary Appendix.

### Cell viability

100 *μ*l of 4×10^4^/ml cell suspension (MDA-MB-231, MCF-7, HeLa, SK-OV-3) was placed in each well of the 96-well culture plate and allowed to grow in dulbecco's modified eagle medium or Leibovitz medium (L-15) supplemented with 10% fetal bovine serum overnight. The cells were incubated with serial dilution of compounds at 310 K in 5% fetal bovine serum containing media for 24 h supplied with 5% CO_2_. At the end of the compounds exposure, 20 *μ*l of 3-(4,5-dimethyl-2-thiazolyl)-2,5-diphenyl-2-H-tetrazolium bromide reagent was added and incubated at 310 K for 4 h. The absorbance of formazan product was measured at 490 nm by a microplate reader (PerkinElmer, Waltham, MA, USA). Cells without peptide were treated as control. *P<0.05, **P<0.01, ***P<0.001 compared with vehicle-treated cells. In Z-VAD-FMK assays, *P<0.05, **P<0.01, ***P<0.001 compared with cells without treatment of Z-VAD-FMK.

### Circular dichroism spectroscopy

Circular dichroism spectra were acquired using Chirascan (Applied Photophysics, Leatherhead, UK) plus circular dichroism spectrometer equipped with a temperature controller using 1 mm cell at a scan speed of 20 nm/s at indicated temperature. Peptide samples were dissolved in phosphate buffer saline (10 mM, pH 7.4, 298 K) with the final concentration between 0.05 and 1 mM. Each sample was scanned twice and the averaged spectrum was smoothed. Final concentrations of the peptides were determined by 280 nm absorption of Trp. Percent helicity was calculated based on the equation: helicity %=[*θ*]_218_/[*θ*]_max_, where [*θ*]_max_=(−44000+ 250T)(1-*k*/*n*) (*T*=298 K, *k*=4.0 and *n*=number of amino acid residues in the peptide).

### Enzyme cleavage assay

For activation of MMPs, MMPs were diluted to 40 *μ*g/ml with 1 mM APMA in assay buffer (50 mM Tris, 10 mM CaCl_2_, 150 mM NaCl, 0.05 (w/v) Brij-35, pH 7.5), and incubated at 310 K for 2 h to activate MMP. Enzymes were then incubated at 50 nM with 3 mM peptide for 30 min at 310 K. Cleavage was performed in a 20 mM Tris buffer with 150 mM NaCl and 2 mM CaCl_2_ at pH 7.4. Samples were diluted into SDS loading buffer, boiled, and run on 15% tricine buffered polyacrylamide gels. Gels were imaged to detect Cy5-labelled peptide.

### Lactate dehydrogenase release

Lactate dehydrogenase release was performed by using Cytotoxicity lactate dehydrogenase release assay Kit-WST (DOJINDO, Kumamoto, Japan). Briefly, 100 *μ*l of 4×10^4^/ml cell suspension was added to each well of the 96-well culture plate and allowed to grow overnight. The cells were then incubated with 50 *μ*l serial dilution of peptides at 310 K for 4 h. Lysis buffer was added as positive control at 310 K for 30 min. Fifty microliter working solution was then added to each well for 30 min at room temperature followed by adding 25 *μ*l stop solution. The absorbance at 490 nm was measured by a microplate reader (PerkinElmer). *P<0.05, **P<0.01, ***P<0.001 compared with corresponding unmasked peptides-treated cells.

### Haemolytic activity

Fresh mouse red blood cells were collected and centrifuged at 800 rpm for 5 min. The erythrocytes were washed and re suspended in 0.9% NaCl to 10^8^/ml. A serial dilution of peptides were added and incubate at 310 K. After 1 h incubation, erythrocytes were centrifuged and the release of hemoglobin was monitored by measuring the absorbance of supernatant at 570 nm by a microplate reader (PerkinElmer). 0.1% Triton X-100 and 0.9% NaCl were employed as positive and negative controls. *P<0.05, **P<0.01, ***P<0.001 compared with corresponding unmasked peptides-treated cells.

### Caspase-3/7 activation analysis

Cells (MDA-MB-231) were plated 10 000 per well on 96-well plates in medium containing 10% fetal bovine serum and incubated with serial dilution of peptides and ABT-737 for 4 h. Then caspase-3/7 activation was measured by addition of the caspase-Glo 3/7 chemiluminescence reagent in accordance with the manufacturer’s protocol (Promega, Madison, WI, USA). Luminescence was detected by a microplate reader (PerkinElmer). *P<0.05, **P<0.01, ***P<0.001 compared with ABT-737-treated cells.

### Xenograft tumour model

Four-week-old nude mice were purchased from the Vital River (Beijing, China) and allowed to grow for further two weeks. Approximately 1×10^6^ MDA-MB-231 cells were injected into the mammary fat pads of female mice in a vehicle of 4 mg/ml Matrigel (Becton Dickinson and Co., New York, NY, USA). One week later, mice bearing tumours around 50 mm^3^ in volume were randomly divided into groups for subsequent experiments.

For optical imaging, mice were anesthetized with isoflurane. Cy5-labelled peptides were injected intravenously at a dose of 100 *μ*l, 50 *μ*M. At each time point, mice were again anesthetized and imaged using IVIS Spectrum Pre-clinical *In Vivo* Imaging System (640 nm/680 nm). Fluorescence in manually set regions of interest was quantified by Living Image. The contrast ratio was calculated by dividing the tumour fluorescence by the chest and abdomen fluorescence. The values for each animal were averaged. *P<0.05, **P<0.01, ***P<0.001 compared with corresponding unmasked peptides-treated mice.

In acute toxicity studies, 12 mice were divided into four groups (*n*=3 for each group) and administered via intravenous injection with peptide (e8-Uk14, e7-Ur11) at the dose of 20 or 40 mg/kg. In chronic renal toxicity studies, mice were administered via intravenous injection with peptide (e8-Uk14, e7-Ur11) at the dose of 20 mg/kg every other day over a periods of 2 weeks.

In tumour growth inhibition studies, mice (*n*=5 for each group) were administered via intravenous injection vehicle control solvent (saline) and peptide at the dose of 20 mg/kg every other day. Tumour size was monitored and measured by caliper measurements over a period of 2 weeks. The volume was calculated using the formula: *V*=1/2 (length) * (width)^2^. Tumour xenografts were excised, routine fixed, paraffin-embedded and sliced for hematoxylin-eosin staining. *P<0.05, **P<0.01, ***P<0.001 compared with vehicle peptides-treated mice.

The animal experiments complied to the Regulations of Guangdong Province on the Administration of Laboratory Animals.

### Statistical analysis

Statistical comparisons were performed between the control and treatment groups using *t*-test using GraphPad Prism 6.0. *P*<0.05 was considered statistically significant.

## Figures and Tables

**Table 1:** Action of Uk14 and Ur11 peptides on different cancer cell lines following a 24 h treatment

*Peptide*	*IC*_*50*_ (*μM)*	
	*Uk14*	*Ur11*
MDA-MB-231	4.5 (0.9)	8.9 (1.8)
MCF-7	4.7 (2.0)	5.6 (2.6)
HeLa	4.8 (0.2)	5.9 (1.1)
SK-OV-3	5.1 (0.5)	5.1 (0.2)

Values are expressed as the mean (standard deviation). MDA-MB-231, breast cancer cell line; MCF-7, breast cancer cell line; HeLa, cervical cancer cell line; and SK-OV-3, human ovary adenocarcinoma cell line. Corresponding cell viability curves are shown in Supplementary Information.

## References

[bib1] Papo N, Shai Y. Host defense peptides as new weapons in cancer treatment. Cell Mol Life Sci 2005; 62: 784–790. 15868403 10.1007/s00018-005-4560-2 PMC11924429

[bib2] Pennarun B, Gaidos G, Bucur O, Tinari A, Rupasinghe C, Jin T et al. killerFLIP: a novel lytic peptide specifically inducing cancer cell death. Cell Death Dis 2013; 4: e894. 24176852 10.1038/cddis.2013.401 PMC3920952

[bib3] Kaufman HL, Kohlhapp FJ, Zloza A. Oncolytic viruses: a new class of immunotherapy drugs. Nat Rev Drug Discov 2015; 14: 642–662. 26323545 10.1038/nrd4663 PMC7097180

[bib4] Orsolic N. Bee venom in cancer therapy. Cancer Metastasis Rev 2012; 31: 173–194. 22109081 10.1007/s10555-011-9339-3

[bib5] Gajski G, Garaj-Vrhovac V. Melittin: a lytic peptide with anticancer properties. Environ Toxicol Pharmacol 2013; 36: 697–705. 23892471 10.1016/j.etap.2013.06.009

[bib6] Moreno M, Giralt E. Three valuable peptides from bee and wasp venoms for therapeutic and biotechnological use: melittin, apamin and mastoparan. Toxins 2015; 7: 1126. 25835385 10.3390/toxins7041126 PMC4417959

[bib7] Krauson AJ, He J, Wimley WC. Gain-of-function analogues of the pore-forming peptide melittin selected by orthogonal high-throughput screening. J Am Chem Soc 2012; 134: 12732–12741. 22731650 10.1021/ja3042004 PMC3443472

[bib8] Wiedman G, Fuselier T, He J, Searson PC, Hristova K, Wimley WC. Highly efficient macromolecule-sized poration of lipid bilayers by a synthetically evolved peptide. J Am Chem Soc 2014; 136: 4724–4731. 24588399 10.1021/ja500462s PMC3985440

[bib9] Wiedman G, Wimley WC, Hristova K. Testing the limits of rational design by engineering pH sensitivity into membrane-active peptides. Biochim Biophys Acta 2015; 1848: 951–957. 25572997 10.1016/j.bbamem.2014.12.023 PMC4331263

[bib10] Krauson AJ, Hall OM, Fuselier T, Starr CG, Kauffman WB, Wimley WC. Conformational fine-tuning of pore-forming peptide potency and selectivity. J Am Chem Soc 2015; 137: 16144–16152. 26632653 10.1021/jacs.5b10595 PMC4697923

[bib11] Liu Q, Zhao H, Jiang Y, Wu M, Tian Y, Wang D et al. Development of a lytic peptide derived from BH3-only proteins. Cell Death Discovery 2016; 2: 16008. 27551502 10.1038/cddiscovery.2016.8 PMC4979451

[bib12] Kohno M, Horibe T, Haramoto M, Yano Y, Ohara K, Nakajima O et al. A novel hybrid peptide targeting EGFR-expressing cancers. Eur J Cancer 2011; 47: 773–783. 21112771 10.1016/j.ejca.2010.10.021

[bib13] Yang L, Horibe T, Kohno M, Haramoto M, Ohara K, Puri RK et al. Targeting interleukin-4 receptor alpha with hybrid peptide for effective cancer therapy. Mol Cancer Ther 2012; 11: 235–243. 22084165 10.1158/1535-7163.MCT-11-0363

[bib14] Kawamoto M, Horibe T, Kohno M, Kawakami K. HER2-targeted hybrid peptide that blocks HER2 tyrosine kinase disintegrates cancer cell membrane and inhibits tumor growth *in vivo*. Mol Cancer Ther 2013; 12: 384–393. 23358664 10.1158/1535-7163.MCT-12-0357

[bib15] Kikuchi O, Ohashi S, Horibe T, Kohno M, Nakai Y, Miyamoto S et al. Novel EGFR-targeted strategy with hybrid peptide against oesophageal squamous cell carcinoma. Sci Rep 2016; 6: 22452. 26956916 10.1038/srep22452 PMC4796678

[bib16] Gaowa A, Horibe T, Kohno M, Sato K, Harada H, Hiraoka M et al. Combination of hybrid peptide with biodegradable gelatin hydrogel for controlled release and enhancement of anti-tumor activity *in vivo*. J Control Release 2014; 176: 1–7. 24378440 10.1016/j.jconrel.2013.12.021

[bib17] Soman NR, Lanza GM, Heuser JM, Schlesinger PH, Wickline SA. Synthesis and characterization of stable fluorocarbon nanostructures as drug delivery vehicles for cytolytic peptides. Nano Lett 2008; 8: 1131–1136. 18302330 10.1021/nl073290r PMC2710241

[bib18] Yang L, Cui F, Shi K, Cun D, Wang R. Design of high payload PLGA nanoparticles containing melittin/sodium dodecyl sulfate complex by the hydrophobic ion-pairing technique. Drug Dev Ind Pharm 2009; 35: 959–968. 19274512 10.1080/03639040902718039

[bib19] Zetterberg MM, Reijmar K, Pranting M, Engstrom A, Andersson DI, Edwards K. PEG-stabilized lipid disks as carriers for amphiphilic antimicrobial peptides. J Control Release 2011; 156: 323–328. 21903146 10.1016/j.jconrel.2011.08.029

[bib20] Dang YQ, Li HW, Wu Y. Construction of a supramolecular forster resonance energy transfer system and its application based on the interaction between Cy3-labeled melittin and phosphocholine encapsulated quantum dots. ACS Appl Mater Interfaces 2012; 4: 1267–1272. 22356839 10.1021/am3000984

[bib21] Huang C, Jin H, Qian Y, Qi S, Luo H, Luo Q et al. Hybrid melittin cytolytic peptide-driven ultrasmall lipid nanoparticles block melanoma growth *in vivo*. ACS Nano 2013; 7: 5791–5800. 23790040 10.1021/nn400683s

[bib22] LeBeau AM, Brennen WN, Aggarwal S, Denmeade SR. Targeting the cancer stroma with a fibroblast activation protein-activated promelittin protoxin. Mol Cancer Ther 2009; 8: 1378–1386. 19417147 10.1158/1535-7163.MCT-08-1170 PMC3348578

[bib23] Sun D, Sun M, Zhu W, Wang Z, Li Y, Ma J. The anti-cancer potency and mechanism of a novel tumor-activated fused toxin, DLM. Toxins 2015; 7: 423–438. 25658509 10.3390/toxins7020423 PMC4344633

[bib24] Jamdade VS, Sethi N, Mundhe NA, Kumar P, Lahkar M, Sinha N. Therapeutic targets of triple-negative breast cancer: a review. Br J Pharmacol 2015; 172: 4228–4237. 26040571 10.1111/bph.13211 PMC4556464

[bib25] Shuman Moss LA, Jensen-Taubman S, Stetler-Stevenson WG. Matrix metalloproteinases: changing roles in tumor progression and metastasis. Am J Pathol 2012; 181: 1895–1899. 23063657 10.1016/j.ajpath.2012.08.044 PMC3506216

[bib26] Vartak DG, Gemeinhart RA. Matrix metalloproteases: underutilized targets for drug delivery. J Drug Target 2007; 15: 1–20. 17365270 10.1080/10611860600968967 PMC3782085

[bib27] Holle L, Song W, Holle E, Wei Y, Wagner T, Yu X. A matrix metalloproteinase 2 cleavable melittin/avidin conjugate specifically targets tumor cells *in vitro* and *in vivo*. Int J Oncol 2003; 22: 93–98. 12469190

[bib28] Holle L, Song W, Holle E, Wei Y, Li J, Wagner TE et al. *In vitro*- and *in vivo*-targeted tumor lysis by an MMP2 cleavable melittin-LAP fusion protein. Int J Oncol 2009; 35: 829–835. 19724919 10.3892/ijo_00000396

[bib29] Jallouk AP, Palekar RU, Marsh JN, Pan H, Pham CT, Schlesinger PH et al. Delivery of a protease-activated cytolytic peptide prodrug by perfluorocarbon nanoparticles. Bioconjug Chem 2015; 26: 1640–1650. 26083278 10.1021/acs.bioconjchem.5b00246 PMC4740973

[bib30] Jiang T, Olson ES, Nguyen QT, Roy M, Jennings PA, Tsien RY. Tumor imaging by means of proteolytic activation of cell-penetrating peptides. Proc Natl Acad Sci USA 2004; 101: 17867–17872. 15601762 10.1073/pnas.0408191101 PMC539314

[bib31] Aguilera TA, Olson ES, Timmers MM, Jiang T, Tsien RY. Systemic *in vivo* distribution of activatable cell penetrating peptides is superior to that of cell penetrating peptides. Integr Biol 2009; 1: 371–381. 10.1039/b904878b PMC2796831 20023744

[bib32] Nguyen QT, Olson ES, Aguilera TA, Jiang T, Scadeng M, Ellies LG et al. Surgery with molecular fluorescence imaging using activatable cell-penetrating peptides decreases residual cancer and improves survival. Proc Natl Acad Sci USA 2010; 107: 4317–4322. 20160097 10.1073/pnas.0910261107 PMC2840114

[bib33] Olson ES, Jiang T, Aguilera TA, Nguyen QT, Ellies LG, Scadeng M et al. Activatable cell penetrating peptides linked to nanoparticles as dual probes for *in vivo* fluorescence and MR imaging of proteases. Proc Natl Acad Sci USA 2010; 107: 4311–4316. 20160077 10.1073/pnas.0910283107 PMC2840175

[bib34] Whitney M, Savariar EN, Friedman B, Levin RA, Crisp JL, Glasgow HL et al. Ratiometric activatable cell-penetrating peptides provide rapid *in vivo* readout of thrombin activation. Angew Chem Int Ed Engl 2013; 52: 325–330. 23080482 10.1002/anie.201205721 PMC3694763

[bib35] Huang S, Shao K, Liu Y, Kuang Y, Li J, An S et al. Tumor-targeting and microenvironment-responsive smart nanoparticles for combination therapy of antiangiogenesis and apoptosis. ACS Nano 2013; 7: 2860–2871. 23451830 10.1021/nn400548g

[bib36] Crisp JL, Savariar EN, Glasgow HL, Ellies LG, Whitney MA, Tsien RY. Dual targeting of integrin alphavbeta3 and matrix metalloproteinase-2 for optical imaging of tumors and chemotherapeutic delivery. Mol Cancer Ther 2014; 13: 1514–1525. 24737028 10.1158/1535-7163.MCT-13-1067 PMC4051287

[bib37] Ellerby HM, Arap W, Ellerby LM, Kain R, Andrusiak R, Rio GD et al. Anti-cancer activity of targeted pro-apoptotic peptides. Nat Med 1999; 5: 1032–1038. 10470080 10.1038/12469

[bib38] Chen WH, Xu XD, Luo GF, Jia HZ, Lei Q, Cheng SX et al. Dual-targeting pro-apoptotic peptide for programmed cancer cell death via specific mitochondria damage. Sci Rep 2013; 3: 3468. 24336626 10.1038/srep03468 PMC3863817

[bib39] Sugihara K, Kobayashi Y, Suzuki A, Tamura N, Motamedchaboki K, Huang CT et al. Development of pro-apoptotic peptides as potential therapy for peritoneal endometriosis. Nat Commun 2014; 5: 4478. 25047118 10.1038/ncomms5478 PMC4109024

[bib40] Nagaraj R, Balaram P. Alamethicin, a transmembrane channel. Acc Chem Res 1981; 14: 356–362.

[bib41] Karle IL, Balaram P. Structural characteristics of alpha-helical peptide molecules containing Aib residues. Biochemistry 1990; 29: 6747–6756. 2204420 10.1021/bi00481a001

[bib42] Demizu Y, Doi M, Kurihara M, Okuda H, Nagano M, Suemune H et al. Conformational studies on peptides containing alpha,alpha-disubstituted alpha-amino acids: chiral cyclic alpha,alpha-disubstituted alpha-amino acid as an alpha-helical inducer. Org Biomol Chem 2011; 9: 3303–3312. 21437330 10.1039/c0ob01146k

[bib43] Sugahara KN, Teesalu T, Karmali PP, Kotamraju VR, Agemy L, Girard OM et al. Tissue-penetrating delivery of compounds and nanoparticles into tumors. Cancer Cell 2009; 16: 510–520. 19962669 10.1016/j.ccr.2009.10.013 PMC2791543

[bib44] Standley SM, Toft DJ, Cheng H, Soukasene S, Chen J, Raja SM et al. Induction of cancer cell death by self-assembling nanostructures incorporating a cytotoxic peptide. Cancer Res 2010; 70: 3020–3026. 20354185 10.1158/0008-5472.CAN-09-3267 PMC2893556

[bib45] Zhou XR, Zhang Q, Tian XB, Cao YM, Liu ZQ, Fan R et al. From a pro-apoptotic peptide to a lytic peptide: one single residue mutation. Biochim Biophys Acta 2016; 1858: 1914–1925. 27207743 10.1016/j.bbamem.2016.05.012

