# Supplementary material for: The development of activatable lytic peptides for targeting triple negative breast cancer
Source: Cell Death Discov. 2017 Jul 17;3:17037–. doi: 10.1038/cddiscovery.2017.37 (PMC5629628; doi:10.1038/cddiscovery.2017.37)
Supplement: Supplementary Information [file cddiscovery201737-s1.pdf]

Supplementary Information

Contents

Tables and Figures 2

Table S1 2

Table S2 2

Figure S1 2

Figure S2 3

Figure S3 3

Figure S4 3

Appendix 4

Mass spectra data for peptides. 4

High performance liquid chromatography trace and mass spectra of representative peptides. 4

Tables and Figures

Table S1 Conformation constraints, mutations, and truncation of (klaklak)2. Action of peptides on MDA-MB-231 cancer cell lines following a 24 h treatment. Values are expressed as the mean (± standard deviation). U=Aib, α-amino iso butyric acid.

peptide	sequence	IC_{50} (μM)
**ak14**	Ac-w-βA-klaklakklaklak-NH_2	>30
**Uk14**	Ac-w-βA-klUklUkklUklUk-NH_2	4.5 (0.9)
**Uk11**	Ac-w-βA-klUkklUklUk-NH_2	19.6 (1.6)
**Ur14**	Ac-w-βA-rlUrlUrrlUrlUr-NH_2	9.3 (1.3)
**Ur11**	Ac-w-βA-rlUrrlUrlUr-NH_2	8.9 (1.8)
**Ur10**	Ac-w-βA-lUrrlUrlUr-NH_2	>20
**lUk**	Ac-w-βA-llllUUUUkkkkkk-NH_2	>40

Table S2 Summary of acute toxicity in mice. Nude mice were injected with peptide and observed for acute toxicity resulting in rapid death. Mouse survival of immediate period after injection was recorded. (n=3 for each group)

peptide	dose	survived
**e8-Uk14**	20 mg/kg	3/3
	40 mg/kg	3/3
**e7-Ur11**	20 mg/kg	3/3
	40 mg/kg	1/3

Figure S1 Conformation constraints, mutations, and truncation of (klaklak)2. Action of peptides on MDA-MB-231. (24 h treatment) Error bars represent the standard deviation from at least two independent experiments. *p < 0.05, **p < 0.01, ***p < 0.001 compared with vehicle-treated cells.

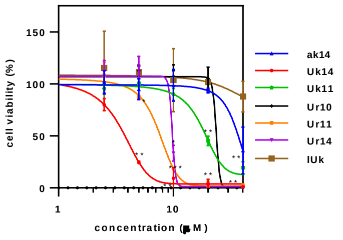

Figure S2 Action of peptides on different cell lines. (24 h treatment) Error bars represent the standard deviation from at least two independent experiments. *p < 0.05, **p < 0.01, ***p < 0.001 compared with vehicle-treated cells.

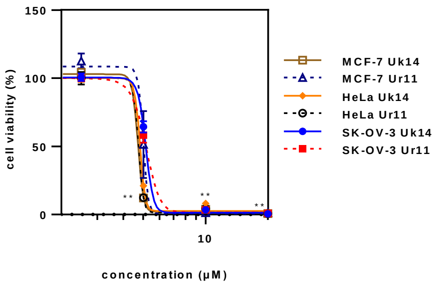

Figure S3 Action of **e8-Uk14** and **e7-Ur11** on HEK293T. (24 h treatment) Error bars represent the standard deviation from at least two independent experiments. *p < 0.05, **p < 0.01, ***p < 0.001 compared with vehicle-treated cells.

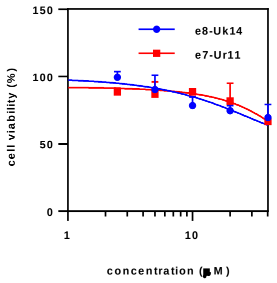

Figure S4 Haematoxylin-eosin staining of the kidney of treated animals. Mice were administered via intravenous injection vehicle control solvent (saline) and peptide at the dose of 20 mg/kg in every other day over a period of 2 weeks. For **e7-Ur11** treated mouse, bleeding was observed at the junction of renal pulp. Scale bar, 300 μm.

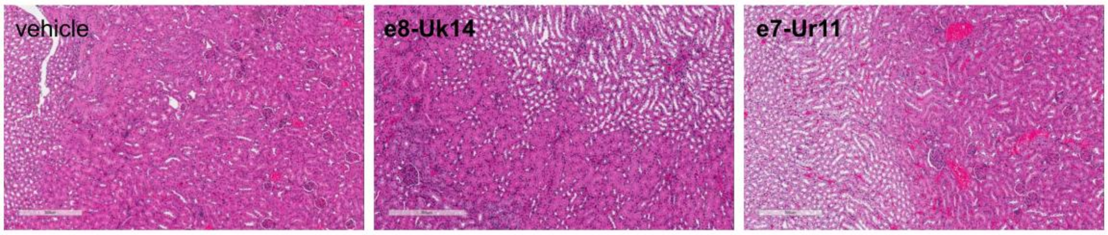

Appendix

Mass spectra data for peptides.

number	sequence	calculated mass	found mass
**ak14**	Ac-w-βA-k-l-a-k-l-a-k-k-l-a-k-l-a-k-NH_2	1821.21	912.0[M+2H]
**Uk14**	Ac-w-βA-k-l-U-k-l-U-k-k-l-U-k-l-U-k-NH_2	1877.27	940.0[M+2H]
**Uk11**	Ac-w-βA-k-l-U-k-k-l-U-k-l-U-k-NH_2	1508.99	776.8[M+2H]
**Ur14**	Ac-w-βA-r-l-U-r-l-U-r-r-l-U-r-l-U-r-NH_2	2045.31	683.1[M+3H]
**Ur11**	Ac-w-βA-r-l-U-r-r-l-U-r-l-U-r-NH_2	1691.07	565.0[M+3H]
**Ur10**	Ac-w-βA-l-U-r-r-l-U-r-l-U-r-NH_2	1534.97	512.9[M+3H]
**lUk**	Ac w-βA-l4-U4-k6-NH_2	1877.27	940.1[M+2H]
**e8-Uk14**	Ac-w-βA-e_8-X-P-L-G-L-A-G- k-l-U-k-l-U-k-k-l-U-k-l-U-k -NH_2	3531.00	1178.6[M+3H]
**e7-Ur11**	Ac-w-βA-e_7-X-P-L-G-L-A-G-r-l-U-r-r-l-U-r-l-U-r-NH_2	3215.75	1073.6[M+3H]
**Uk14**$_{CY5}$	Ac-k-l-U-k-l-U-k-k-l-U-k-l-U-k-k-(Cy5)-NH_2	2387.47	1195.2[M+2H]
**e8-Uk14**$_{CY5}$	Ac-e_8-X-P-L-G-L-A-G-k-l-U-k-l-U-k-k-l-U-k-l-U-k-k-(Cy5)-NH_2	4041.19	1348.7[M+3H]
**e8-Uk14**$_{uc\ CY5}$	Ac-e_8-X_3-k-l-U-k-l-U-k-k-l-U-k-l-U-k-k-(Cy5)-NH_2	3759.06	1254.6[M+3H]
**Ur11**$_{CY5}$	Ac-r-l-U-r-r-l-U-r-l-U-r-k-(Cy5)-NH_2	2201.27	734.9[M+3H]
**e7-Ur11**$_{CY5}$	Ac-e_7-X-P-L-G-L-A-G-r-l-U-r-r-l-U-r-l-U-r-k-(Cy5)-NH_2	3725.95	1243.3[M+3H]

U=Aib, X=6-aminohexanoyl

High performance liquid chromatography trace and mass spectra of representative peptides.

Uk14

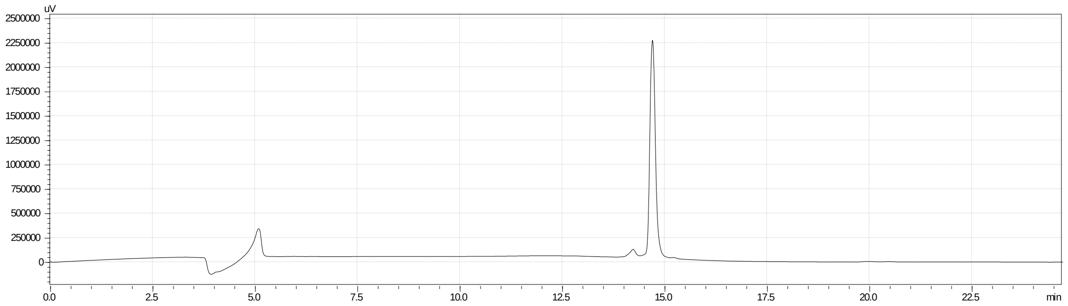

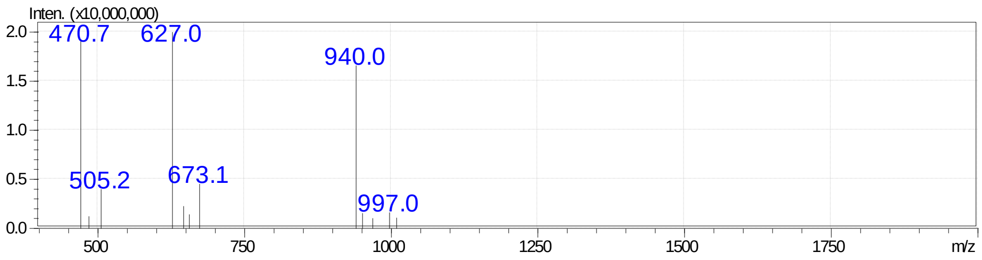

Ur11

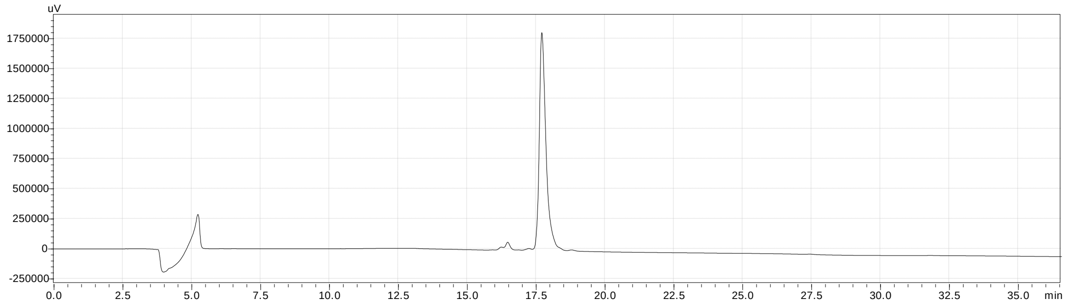
uV
1750000
1500000
1250000
1000000
750000
500000
250000
0
-250000
0.0
2.5
5.0
7.5
10.0
12.5
15.0
17.5
20.0
22.5
25.0
27.5
30.0
32.5
35.0
min

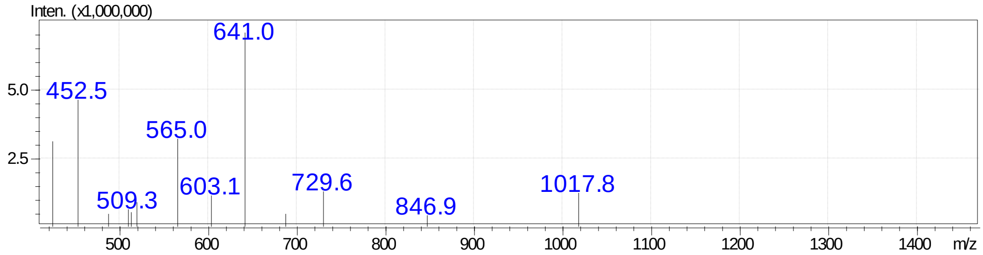
Inten. (x1,000,000)
641.0
452.5
565.0
509.3
603.1
729.6
846.9
1017.8
5.0
2.5
m/z

e8-Uk14

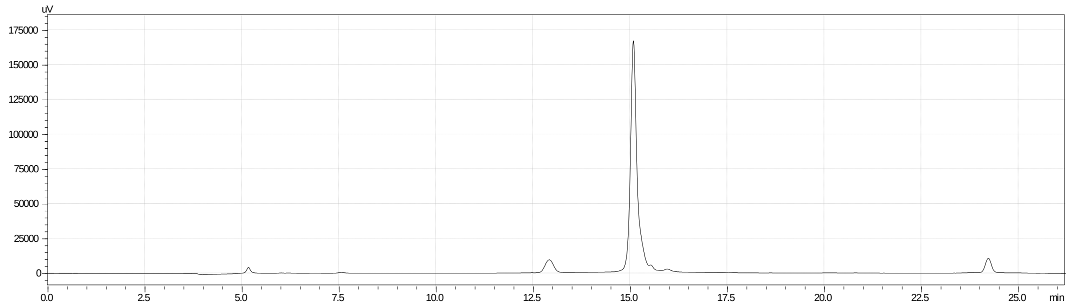
uV
175000
150000
125000
100000
75000
50000
25000
0
0.0
2.5
5.0
7.5
10.0
12.5
15.0
17.5
20.0
22.5
25.0
min

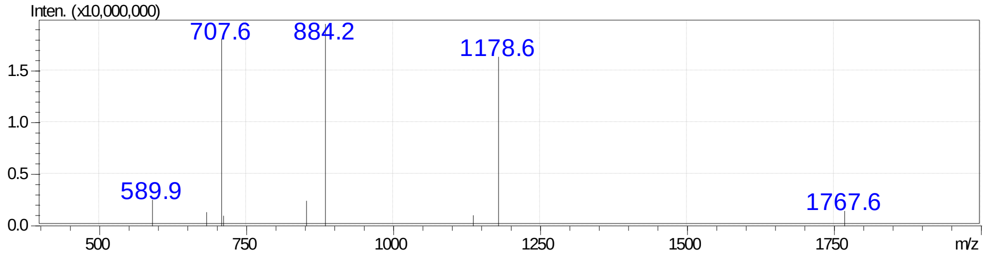
Inten. (x10,000,000)
707.6
884.2
1178.6
589.9
1767.6
1.5
1.0
0.5
0.0
m/z

e7-Ur11

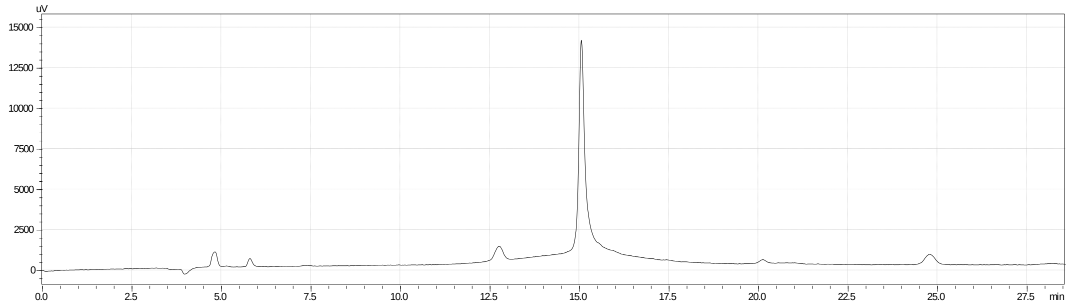

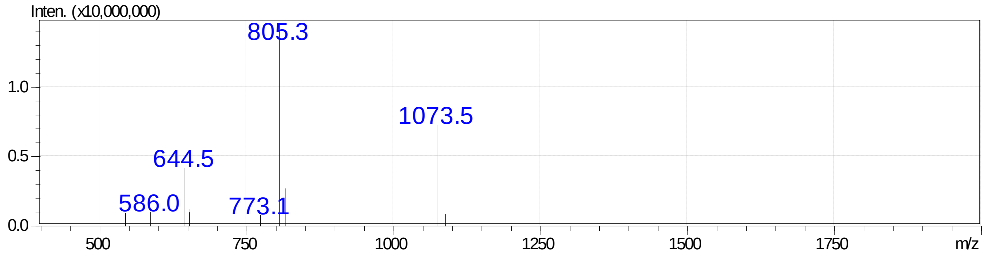

e8-Uk14CY5

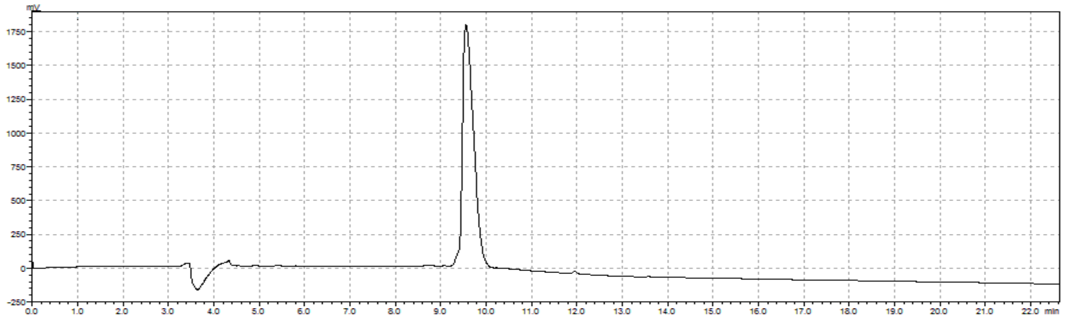

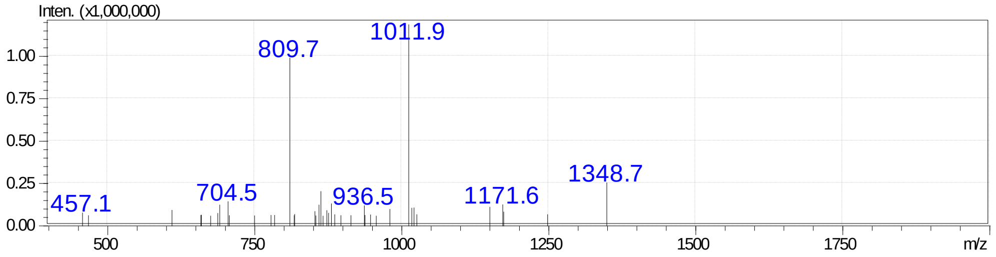

e7-Ur11CY5

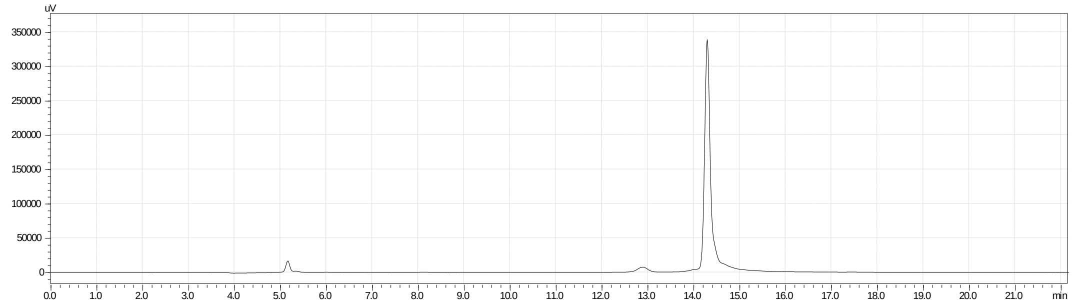
uV
350000
300000
250000
200000
150000
100000
50000
0
0.0 1.0 2.0 3.0 4.0 5.0 6.0 7.0 8.0 9.0 10.0 11.0 12.0 13.0 14.0 15.0 16.0 17.0 18.0 19.0 20.0 21.0 min

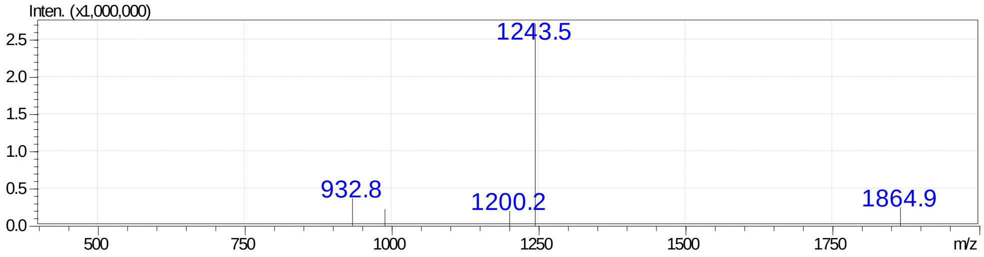
Inten. (x1,000,000)
2.5
2.0
1.5
1.0
0.5
0.0
1243.5
932.8
1200.2
1864.9
500 750 1000 1250 1500 1750 m/z
